# Draft Genome Sequence of *Planomonospora sphaerica* JCM9374, a Rare Actinomycete

**DOI:** 10.1128/genomeA.00779-16

**Published:** 2016-08-04

**Authors:** Hideo Dohra, Tomohiro Suzuki, Yuto Inoue, Shinya Kodani

**Affiliations:** a Instrumental Research Support Office, Research Institute of Green Science and Technology, Shizuoka University, Suruga-ku, Shizuoka, Japan; b Department of Science, Graduate School of Integrated Science and Technology, Shizuoka University, Suruga-ku, Shizuoka, Japan; c Center for Bioscience Research and Education, Utsunomiya University, Utsunomiya, Tochigi, Japan; d Department of Agriculture, Graduate School of Integrated Science and Technology, Shizuoka University, Suruga-ku, Shizuoka, Japan

## Abstract

*Planomonospora sphaerica* is a rare actinomycete that is a potential antibiotic producer. Here, we report the draft genome sequence of *P. sphaerica* strain JCM9374. This is the first genome report of a bacterium belonging to the genus *Planomonospora*. The genome information of *P. sphaerica* will contribute to studies on the structure and function of antibiotics.

## GENOME ANNOUNCEMENT

In a previous study, a rare actinomycete *Planomonospora sphaerica* was reported to show antibacterial activity against *Micrococcus luteus* (1). However, the antibacterial principle has not been identified yet. Microbial genome mining on the basis of next-generation sequencing technology is a powerful approach to discover the antibacterial compounds and biosynthetic pathways. The genome sequence of *P. sphaerica* was determined and the genes possibly involved in the antibacterial activity were searched for.

A genomic DNA of *P. sphaerica* strain JCM9374^T^ was extracted using the DNeasy blood and tissue kit, and then paired-end genome libraries (enzymatic fragmentation for 3 and 5 min) were constructed using the KAPA HyperPlus kit. Genome libraries of *P. sphaerica* were sequenced using the Illumina MiSeq platform at the Instrumental Research Support Office, Research Institute of Green Science and Technology, Shizuoka University. Paired-end raw reads (101-bp) were cleaned using Trimmomatic (2) by trimming adapter sequences and low-quality ends (quality score, <15) and khmer (3) by filtering the reads with a low k-mer coverage (<5) to remove sequence errors and contamination sequences. The resultant 1,305,056 high-quality reads totaling 611 Mb, which corresponds to an approximately 75-fold coverage of the genome, were assembled using SPAdes version 3.6.2 (4) with a k-mer size of 21, 33, 55, 77, 99, and 127 bp with the careful option, and contigs less than 200-bp were eliminated. The draft genome of *P. sphaerica* JCM9374 contains 84 contigs consisting of 8,132,638 bp with a G+C content of 72.73%.

The draft genome sequence was annotated using Prokka version 1.11 (5) with an in-house bacterial database containing 5,399 bacterial genomes (chromosomes and complete genomes only) in the NCBI RefSeq database as of 8 April 2016, and tRNA genes were predicted using tRNAscan-SE version 1.3.1 (6). The annotated genome contains 7,166 protein-coding sequences and 61 tRNA genes. The use of the in-house bacterial database reduced the number of proteins annotated as “hypothetical proteins” compared to the original database included in the Prokka (from 2,146 to 1,198), which provides more information for prediction of the protein function. In the genome of *P. sphaerica*, we found genes encoding proteins possibly involved in antibacterial activity, such as non-ribosomal peptide synthetases, bacteriocin, and lantibiotic biosynthesis proteins. The proteins encoded by the genome were additionally annotated with the COG (Clusters of Orthologous Groups) database (7) and Pfam database of protein families (8). Among 7,166 proteins, 4,843 (67.6%) and 5,570 (77.7%) were assigned to the COG functional categories and Pfam protein families, respectively, including siderophore synthetase component (COG4264), lanthionine synthetase C-like protein (PF05147), and lantibiotic dehydratase, C-terminus (PF04738). The genome information of *P. sphaerica* will contribute to studies on the structure and function of antibacterial peptides and the discovery of novel bioactive compounds and biosynthetic pathways.

### Accession number(s).

The draft genome sequence of *P. sphaerica* strain JCM9374 has been deposited in the DDBJ/EMBL/GenBank under the accession no. BDCX00000000.
